# Jaw Pain and Profound Bradycardia – An Atypical Presentation of Lyme Carditis

**DOI:** 10.7759/cureus.11607

**Published:** 2020-11-21

**Authors:** Samuel Schick, Ryan Quigley, Zachary A Koenig, Ryan McCarthy

**Affiliations:** 1 School of Medicine, West Virginia University, Morgantown, USA

**Keywords:** lyme carditis, borrelia burgdorferi, bradycardia

## Abstract

In 2018, 23,558 confirmed cases and 10,108 probable cases of Lyme disease were reported in the United States, with 96% of all cases coming from 14 states. Lyme carditis is well described, occurring in less than 1% of Lyme disease. High-grade heart block is uncommon in early disseminated Lyme disease. In Lyme carditis due to sinus node dysfunction and/or high grade atrioventricular block, the pulse rates are significantly lower which can lead to syncope. This can happen in the setting of an unstable ventricular escape rhythm with pulse rates ranging around 30 beats per minute or lower. In patients with low cardiovascular reserve, high-degree AV block can cause sudden death. Here we describe a rare case of profound bradycardia in disseminated Lyme disease. The patient’s only two symptoms are bradycardia and jaw pain. He lacks erythema migrans, neurological symptoms or syncope - despite having high-degree AV block. Initially prescribed doxycycline 100mg BID, his PR interval begins to normalize, but once a Lyme titre was positive for IgM (p41, p39, p23) and IgG (p66, p45, p41, p39, p23, p18), the patient was switched to 2g ceftriaxone IV Q 24h, per Infectious Disease Society of America (IDSA) guidelines. After several days he feels better and was discharged home to complete antibiotics and wear a cardiac event monitor. Lyme disease has three distinct stages that include early localized infection, early disseminated disease, and late infection. At the time of Lyme carditis diagnosis, common symptoms include erythema migrans, malaise, polyarthritis, Bell’s palsy and other neurological symptoms - all of which were lacking in our patient. The prognosis for Lyme carditis is generally good, despite disagreement over the incidence of persistent B. burgdorferi infection. This patient’s unique presentation of Lyme carditis is further evidence of variability in cardiac symptoms depending on one’s immunological and physiological ability to combat acute spirochete infection.

## Introduction

Lyme carditis most commonly presents as high-degree AV block and is well described, occurring in less than 1% of Lyme disease cases [1]. Borrelia burgdorferi is a spirochete transmitted by the bite from I. scapularis tick [2]. Infection rates in the United States have risen in recent years. Concentrated in the northeast, 14 states make up more than 96% of all cases. In 2000, 17,730 cases were reported in 44 states [3]. In 2018, 23,558 confirmed cases and 10,108 probable cases were reported. Most cases of Lyme disease occur between June and December, with bimodal peak incidence between 5- to 14-year-olds and 44- to 59-year-olds [4].

B. burgdorferi infection presents with a variety of symptoms: fever, malaise, erythema migrans, arthritis [5]. Due to increased awareness and treatment of Lyme disease, the prevalence of disseminated spirochetal infection and advanced stage symptoms has decreased in recent decades. Lyme carditis is a hallmark of early disseminated disease and is estimated to arise in roughly 1% of all cases of recognized infections [6,7]. Patients can develop heart block which often presents as syncopal episodes, or sudden death [8]. Bell’s palsy has been reported in 8% of untreated patients [7,9]. Progression from initial infection to Lyme carditis was found to occur 21 days after onset of erythema migrans [5]. B. burgdorferi infection is often a clinical diagnosis solely based on physical exam: erythema migrans, the classic target rash surrounding the ixodes tick bite [9]. While this physical exam finding is often considered by clinicians to be ubiquitous, it only occurs in about 70 to 80 percent of cases [6]. Given the potential for severe manifestations of untreated B. burgdorferi infection, timely recognition and treatment is essential to prevent disseminated disease.

## Case presentation

A 40-year-old male with tachycardia and left-sided jaw pain radiating to his chest presented to the ED. Electrocardiogram (EKG) showed sinus tachycardia (103 BPM). Troponin was slightly elevated at initial presentation (0.08 ng/mL) and downward trending in subsequent measurements. Brain natriuretic peptide (BNP) was 300 pg/mL. The patient was admitted to the hospital to rule out acute coronary syndrome (ACS). A treadmill cardiac stress test was negative for ischemia. Transthoracic echocardiogram (TTE) was within normal limits, with an ejection fraction of 56% and no wall motion abnormalities.

**Figure 1:**
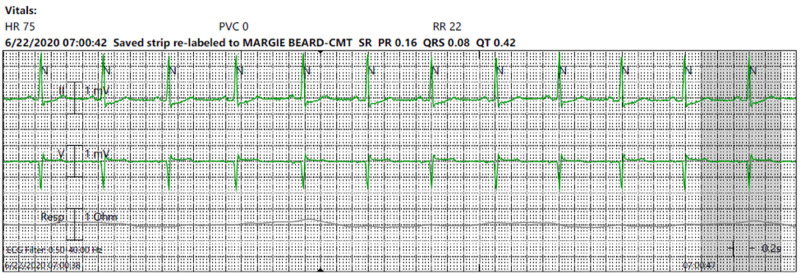
Electrocardiogram (ECG) four weeks prior to Lyme carditis diagnosis.

The patient achieved an METs score of 17. TTE and chest X-ray showed no abnormal findings. The patient received a general diagnosis of unexplained cardiac changes due to stress and was discharged home. While visiting his primary care physician (PCP) to discuss the isolated findings of jaw pain and mildly elevated troponin, the patient was noted to have sinus conduction delay on EKG, with a PR-interval of 160 milliseconds (Figure 1).

Two weeks after the occurrence of jaw pain, the patient was roused from sleep by dyspnea and back pain. At the time, the patient attributed the back pain to sprain from exercise. The patient noted that during this episode of his pulse was very low, recorded at 26 bpm and occasionally undetectable upon radial artery palpation.

Table 1 illustrates progressive PR-interval prolongation and subsequent resolution following antibiotic administration. Additionally, slight elevation of troponin is suggestive of some degree of myocardial necrosis resulting from infection.

**Table 1:** Various cardiac parameters after initial symptom onset on 6/22.

	6/22/20	7/14/20	7/27/20	7/27/20	7/28/20	8/7/20
Ventricular rate (bpm)	103	58	76	66	56	83
Atrial rate (bpm)	103	58	76	66	56	83
PR interval (ms)	148	400	300	290	250	198
QRS duration (ms)	84	88	84	84	80	86
QT interval (ms)	328	446	494	414	432	392
QTC calc.	429	437	454	434	416	460
P axis		43	60	62	44	75
R axis	-116	77	73	64	73	74
T axis	-45	-29	-43	RR	29	29

**Figure 2:**
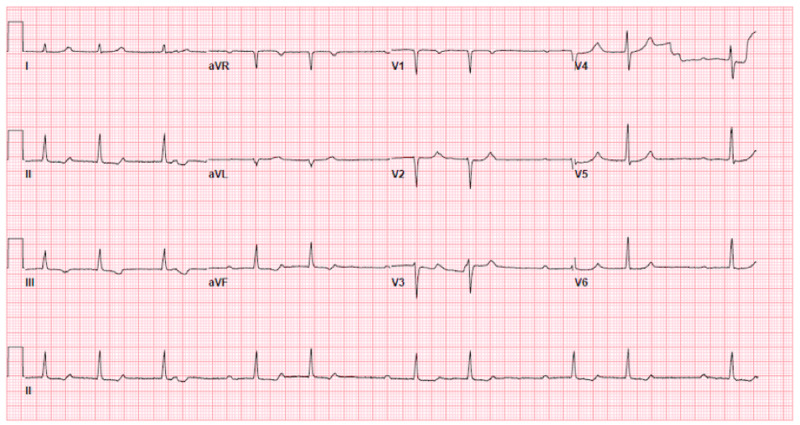
Second degree AV block recorded one month after normal ECG tracing in Figure 1.

Over the course of the next month several EKGs would note the patient’s PR-interval was between 250ms, 1st degree AV block, and 400ms, 2nd degree AV block (Figure 2). The patient noted migratory arthralgia intermittently in the week prior to diagnosis of Lyme carditis. Additionally, the patient admitted to experiencing some degree of anterior lymphadenopathy of the neck in the day leading up to diagnosis. A month after the initial presentation, an EKG at the cardiologist office showed a rate of 58 bpm and a 2nd-degree AV block Mobitz type I.

The patient was prescribed doxycycline 100mg BID, after which his PR interval began to decrease. A Lyme titre was checked using immunohistochemical markers and was positive for IgM (p41, p39, p23) and IgG (p66, p45, p41, p39, p23, p18). Per Infectious Disease Society of America (IDSA) guidelines, the patient was switched to 2g ceftriaxone IV every 24h once suspected Lyme carditis was confirmed by immunohistochemical testing. After several days he reported feeling better and was discharged with the remaining course of antibiotics and a portable cardiac event monitor.

## Discussion

Clinical manifestations

Lyme disease presents in three distinct stages that include early localized infection, early disseminated disease, and late infection. Early localized infection of Lyme disease occurs 7-10 days after a tick bite and the most typical presentation is a single annular rash expanding from the area of the bite. This stage is without signs of systemic involvement.

Early disseminated disease occurs weeks to months later; patients can have multiple expanding annular lesions, lymphadenopathy, migratory joint and muscle pain, meningitis with cranial nerve involvement, and Lyme carditis [5]. At the time of Lyme carditis diagnosis, common symptoms attributable to the pathogen include erythema migrans, malaise, polyarthritis, Bell’s palsy and, less commonly, severe neurological symptoms such as encephalitis.

Among specific cardiac manifestations of Lyme disease, myocarditis is relatively rare [10]. Infection of the myocardium by B. burgdorferi typically presents 1-2 months after onset of infection, and while endocardial biopsy would have been confirmatory of this in this case, the invasiveness of the procedure relegates this diagnostic tool to a matter of academic curiosity. Without practical methods to quantify myocardial involvement on EKG, it is possible that the incidence of myocarditis is higher.

Serology

The Centers for Disease Control and Prevention (CDC) recommends a two-tiered strategy for serological identification, whereby a negative ELISA is followed up a month later with another ELISA or confirmatory Western Blot if two of three IgM (21-24, 39, and 41 kDa) or five of 10 IgG bands (18, 21-24, 28, 30, 39, 41, 45, 58, 66, and 93 kDa) are positive on initial ELISA testing [11]. In order to test for serology, Lyme disease must be included in the list of suspected causes of high degree AV block in patients presenting similar to the aforementioned case. The suspicious index in Lyme carditis score evaluates the likelihood that a patient’s high-degree atrioventricular block is caused by Lyme carditis (Table 2). The total summed score indicates low (0-2), intermediate (3-6), or high (7-12) suspicion of Lyme carditis.

**Table 2:** Suspicion index for Lyme carditis (SILC). Adapted from Besant et al. [12].

Variable	Points
Sex = male	1
Age < 50	1
Constitutional symptoms (fever, arthralgia, dyspnea)	2
Tick bite	3
Erythema migrans	4

Diagnostic criteria

At initial presentation, with an age of 41 and male sex, and no known history of a tick bite or erythema migrans, the patient discussed above received a SILC score of 2, and was considered low suspicion for Lyme carditis. During the following four weeks, nonspecific symptoms of arthralgia increase the SILC score to 4, or intermediate. EKG findings of PR-interval prolongation are nonspecific, but helpful in monitoring progression and recovery. Imaging findings were unremarkable, and thus do not play a major role in identification of Lyme carditis in this patient. Case reports have shown that some nonspecific cardiac changes can be seen on MRI, such as slight left ventricular dilation and mitral regurgitation as well as persistent pericardial signal change [13].

Treatment

Significant disagreement exists regarding the treatment of Lyme carditis, with multiple schools of thought surrounding the possibility of persistent infection following short treatment regimen (Table 3) [14]. However, early recognition and treatment is generally accepted as beneficial, with additional reduction in mortality from temporary implantation of a cardiac pacemaker to prevent progression from high degree AV-block to asystole during initial treatment.

**Table 3:** Lyme carditis treatment options. Adapted from Grella et al. [11].

Centers for Disease Control and Prevention (CDC)	Doxycycline 100 mg orally twice daily for 10-21 days or Cefuroxime Axetil 500 mg orally twice daily for 14-21 days or Amoxicillin 500 mg orally three times daily for 14-21 days
International Lyme and Associated Diseases Society (ILADS)	Amoxicillin 1500-2000 mg orally daily in divided doses for 4-6 weeks or Cefuroxime 500 mg orally twice daily for 4-6 weeks or Doxycycline 100 mg orally twice daily for 4-6 weeks or Azithromycin 250-500 mg orally daily for 21 days
Infectious Disease Society of America (IDSA)	Preferred Treatment: Ceftriaxone 2 grams once per day via IV for 14 days with a range of 10-28 days. Alternative Treatments: Cefotaxime 2 grams IV every 8 hours or Penicillin G 18-24 million units per day in patients with normal renal function divided into doses given every 4 hours or Doxycycline 200-400 mg per day in 2 divided doses orally for 10-28 days for patients intolerant of B-lactam antibiotics

Prognosis

The prognosis for Lyme carditis is generally good, despite disagreement over the incidence of persistent B. burgdorferi infection that is a subject of extensive debate. Indirect measure of recovery through decrease in PR-interval shows varying speed of recovery from high degree AV block, ranging from weeks to months for recovery of normal 200 ms conduction timing in healthy individuals [15].

## Conclusions

Despite low-to-intermediate SILC score, the patient’s progressive AV block due to Lyme carditis is an atypical presentation of Lyme disease. This patient's presentation is further evidence that the spectrum of symptomology varies considerably across organ system with variation of cardiac-specific symptomology possibly dependent on a combination of immunological and physiological ability to combat the spirochetal infection.
